# Pyroglutamic acidosis in the setting of *Staphylococcus aureus* bacteremia treated with flucloxacillin—An uncommon cause of high anion gap metabolic acidosis

**DOI:** 10.1002/ccr3.9243

**Published:** 2024-08-07

**Authors:** Aaron Yee Shuen See, Ashton Zheng‐Boon Lau, Srivathsan Thiruvengadam

**Affiliations:** ^1^ Medical School University of Western Australia Perth Western Australia Australia; ^2^ Internal Medicine Unit Royal Perth Hospital Perth Western Australia Australia

**Keywords:** general medicine, medical education, nephrology, pharmacology and pharmacy, physiology

## Abstract

Pyroglutamic acidosis (PGA) or 5‐Oxoprolinuria is an uncommon and often overlooked cause of high anion gap metabolic acidosis (HAGMA). This case highlights the importance of systematically approaching HAGMA, and to consider PGA as a differential diagnosis when medications that disrupt the γ‐glutamyl cycle such as flucloxacillin and paracetamol are present.

## 1 INTRODUCTION

**FIGURE 1:**
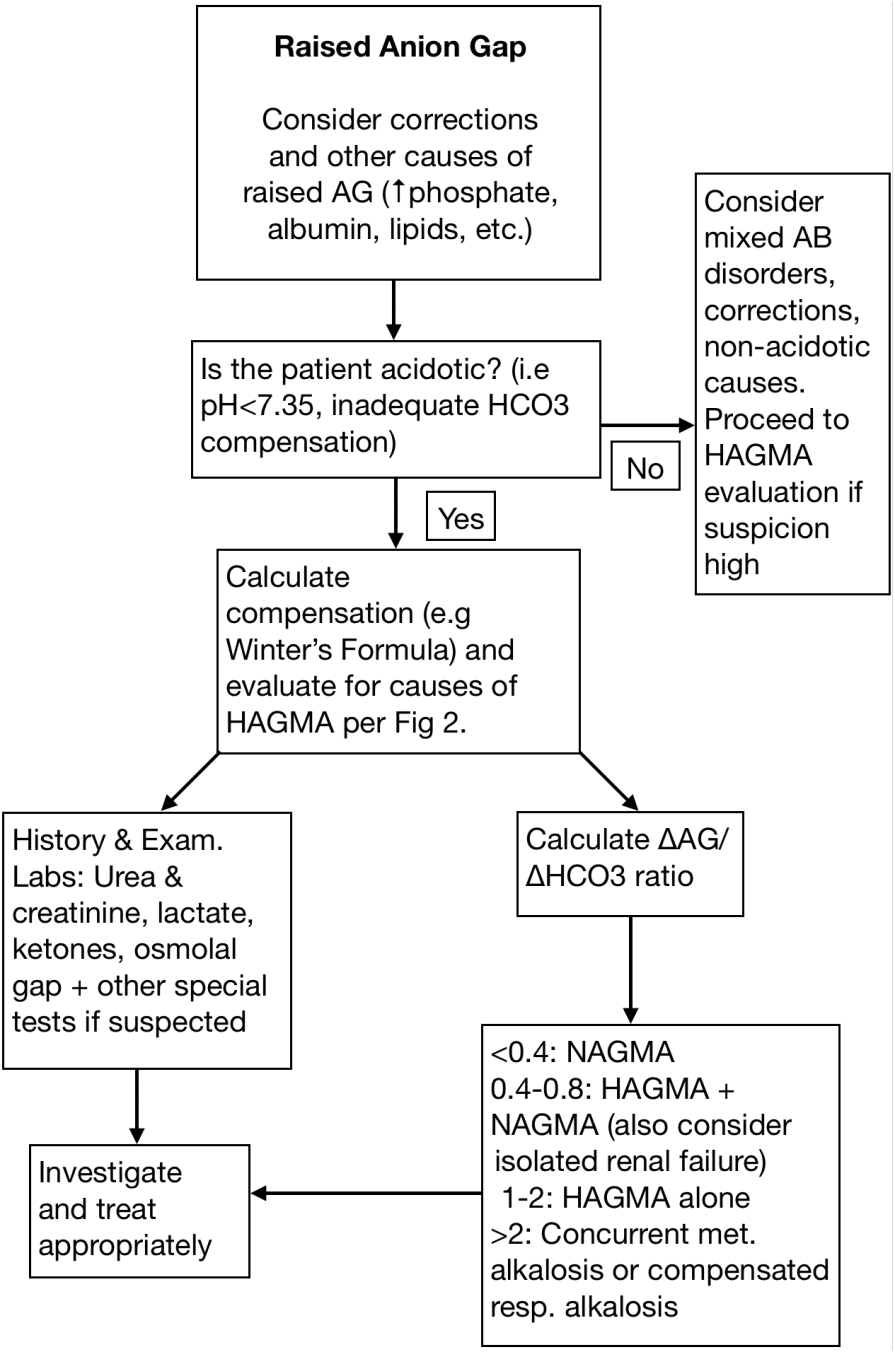
Workup of HAGMA. AB, acid–base; AG, anion gap; HAGMA, high anion gap metabolic acidosis; met., metabolic; NAGMA, non‐anion gap metabolic acidosis; resp., respiratory.

**FIGURE 2:**
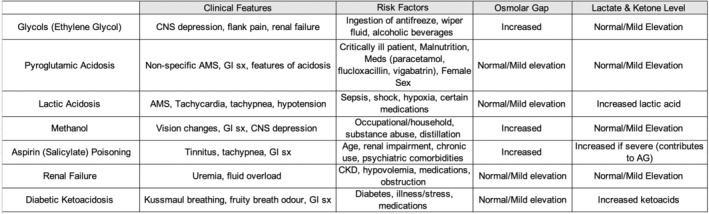
Differentials for HAGMA. AG, anion gap; AMS, altered mental status; CKD, chronic kidney disease; CNS, central nervous system; GI, gastrointestinal; sx, symptoms.

Metabolic acidosis is a common acid–base derangement which can arise from various causes and requires a structured approach. Metabolic acidosis is commonly categorized into normal or high anion gap metabolic acidosis based on the levels of unmeasured anions in the blood. Causes of HAGMA include glycols, 5‐oxoproline, l‐lactate, d‐lactate, methanol, aspirin, renal failure, and ketoacidosis—or “GOLDMARK”.^1^ The evaluation of a raised anion gap begins with the determination of acid–base status and compensation (Figure 1). Even in the absence of acidosis, a raised anion gap can still be suggestive of an underlying HAGMA in cases of mixed acid–base disorders and non‐acidotic causes such as hyperphosphatemia and hyperlipidemia.^2^ It is therefore important to calculate compensation, take a thorough history and examination, and order relevant investigations such as lactate, ketones, osmolal gap, and other relevant laboratories to further evaluate the cause (Figure 2). Calculating the ΔAG/ΔHCO_3_ ratio to assess for concurrent acid–base disorders is also an important aspect of HAGMA workup.^2^

Pyroglutamic acidosis (PGA), caused by the metabolic accumulation of 5‐oxoproline, is an important differential to consider in HAGMA. This report describes a case of PGA secondary to methicillin‐sensitive Staphylococcus aureus (MSSA) bacteremia and osteomyelitis treated with flucloxacillin. The condition manifested as a notable, asymptomatic decrease in bicarbonate following commencement of flucloxacillin. Urine pyroglutamic acid levels confirmed the diagnosis, and the patient was treated with discontinuation of offending medications and oral bicarbonate.

## 2 CASE HISTORY/EXAMINATION

A 64‐year‐old male presented to the emergency department with left sided neck pain associated with headache, dizziness, vomiting, and rigors. The neck pain, which did not radiate but worsened on movement, developed spontaneously 5 days ago without any precipitating factors, including recent trauma or illness. The pain persisted, and on the day of presentation, the patient developed sudden onset nausea, vomiting, dizziness, diarrhea, and rigors, which prompted him to seek medical attention. The patient otherwise denied any fever, shortness of breath, cough, chest pain, syncope, vision changes, weakness, or urinary symptoms. His background history included type II diabetes mellitus complicated by Charcot arthropathy, epilepsy, benign prostatic hyperplasia, and psoriasis. Additionally, the patient has mild intellectual impairment and was under the care of a public guardian.

The patient's medications included levetiracetam, phenytoin, paracetamol, metformin, dulaglutide, glargine, gliclazide, thiamine, prazosin, mirtazapine, pantoprazole, cholecalciferol, and fexofenadine. On examination, the patient was alert and oriented; vital signs showed heart rate of 73 bpm, blood pressure of 100/70 mmHg, respiratory rate of 16 breaths/minute, O_2_ saturation of 98% on room air, and temperature of 36.3°C. Point tenderness was noted on palpation of the cervical spine, and pain was elicited in the left hip and knee on passive movement. A splinter hemorrhage on his right fifth digit was noted. On auscultation, he had bilateral vesicular breath sounds and dual heart sounds without any murmurs. Laboratory results (Table 1) were notable for a raised C‐reactive protein (CRP) of 215 mg/L; full blood count, serum electrolytes, urea, and creatinine were unremarkable. The patient was admitted to hospital for further evaluation.

**TABLE 1:** Laboratory results and trends.

	Initial hospitilization	After 2 weeks treatment	Post drug cessation	Reference range
Sodium	134	138	139	135–145 mmol/L
Chloride	103	110	108	96–106 mmol/L
Potassium	4.1	4.1	4.1	3.5–5.0 mmol/L
Bicarbonate	23	12	23	22–32 mmol/L
pH	7.4	7.28	7.33	7.32–7.43
pco_2_	35	34	44	36–44 mmHg
Albumin	34	27	31	32–45 g/L
Anion gap (corrected)	8.0 (9.5)	15 (19)	8 (10.3)	4–12 mmol/L
ΔAG/ΔHCO_3_	–	0.61	–	See Figure 1
Urea	6.2	4	4.1	3–10 mmol/L
Creatinine (Cr)	49	51	44	60–110 μmol/L
Lactate	<1.5	<1.5	<1.5	<1.5 mmol/L
Ketones	<0.6	<0.6	<0.6	<0.6 mmol/L
Urine 5‐oxoproline	–	8.5	<0.1	<0.1 mmol/mmol Cr
Serum osmolality	–	287	–	275–295 mOsm/kg
C‐Reactive protein	215	43	75	<5 mg/L

## 3 METHODS

Given the presentation of localized neck pain and sudden onset of headache, dizziness, and vomiting, there was immediate concern for vertebral artery dissection. Cervical spine pathology such as osteomyelitis, discitis, spinal abscess, and pathological fractures were also considered given the reported rigors, localized tenderness, patient's age, and history of complicated diabetes. A CT angiogram head and neck with contrast was subsequently performed for suspected vertebral artery dissection and secondary evaluation of cervical spine. Although no dissection was identified, a C3 wedge fracture was noted. Due to the acute and atraumatic nature of the fracture, an MRI of the neck was performed, which demonstrated enhancing lesions concerning for metastases. This was investigated further with a CT of his chest, abdomen, and pelvis, which did not reveal any sources of malignancy. However, a gallium bone scan was subsequently performed due to persisting CRP elevation, which demonstrated hypervascularity and increased uptake in the C3 region, left periarticular hip structures, and hand digit. In addition, the patient's blood and urine culture returned positive for MSSA. A protective cervical collar was placed, and the patient was started on IV flucloxacillin 2 g QID for presumptive MSSA bacteremia, osteomyelitis, septic arthritis, and bacteriuria from presumed hematogenous spread.

A consultation to the infectious disease team was undertaken. It was decided that the patient needed a total of 6 weeks of IV flucloxacillin treatment. Following 2 weeks of flucloxacillin treatment, there was a noticeable drop in bicarbonate levels from a stable baseline average of 23 mmol/L to 12 mmol/L [22–32 mmol/L]. Sodium was 138 mmol/L [135–145 mmol/L], chloride 110 mmol/L [96–106], and potassium 4.1 mmol/L [3.5–5.0 mmol/L]. Creatinine, urea, and ketone levels were within normal limits. The patient was otherwise asymptomatic with no medical complaints, although the patient had mild malnutrition secondary to multifactorial prolonged hospitalization, ongoing infection, and difficulty eating with cervical collar. Vital signs and physical examination were unremarkable. A venous blood gas was subsequently performed, which showed a pH of 7.28 [7.32–7.43], pco_2_ of 34 [36–44 mmHg], lactate of 1.0 [<1.5 mmol/L]. When corrected for a serum albumin of 27 [32–45 g/L], the anion gap (AG) was elevated at 19.3 [4–12 mmol/L]. Given the raised anion gap and pattern of acidosis, HAGMA was suspected. pco_2_ was near compensated (29–33 mmHg), and the calculated ΔAG/ΔHCO_3_ of 0.61 was concerning for a mild concurrent non‐anion gap metabolic acidosis. Investigating for likely causes of HAGMA, clinical suspicion for lactic acidosis was low based on a normal lactate level along with absent features of sepsis and normal vital signs. Suspicion for renal failure and ketoacidosis was also low based on examination and negative laboratory findings. In addition, given the patient's prolonged hospital stay, lack of suggestive clinical features such as visual/neurological changes, and normal serum osmolality, suspicion for toxic alcohol ingestion was also low. Considering the unexplained drop in bicarbonate following administration of IV flucloxacillin in a patient with chronic paracetamol use and multiple medical conditions, the suspicion for pyroglutamic acidosis was high. This was confirmed with a urine organic acid profile, which demonstrated a markedly elevated 5‐oxoproline level of 8.5 mmol/mmol creatinine [<0.1], confirming the diagnosis of PGA. In terms of the concurrent NAGMA, the absence of certain medications and history suggested the cause as likely secondary to IV fluid, nutritional support, gastrointestinal loss, and malnutrition. No further workup was done for renal tubular acidosis.

## 4 RESULTS

Following identification of the cause of HAGMA, the flucloxacillin dose frequency was reduced and paracetamol 500 mg PRN was immediately ceased. However, the anion gap metabolic acidosis persisted, with bicarbonate reaching a nadir of 11 mmol/L. The flucloxacillin was subsequently swapped to IV cefazolin 2 g TDS. In addition, oral bicarbonate was administered due to the low bicarbonate level and the patient had repeat venous blood gas measurements over the following days. Resolution of acidosis was confirmed 4 days later, with bicarbonate levels returning to pre‐treatment baseline level of 23 mmol/L.

Following the resolution of acidosis, the patient had an uncomplicated stay and was stepped down to PO Cefalexin 1 g QID after 6 weeks of IV antibiotic therapy. Blood and urine cultures were subsequently negative, and repeat MRI showed expected progression of inflammatory changes. The patient was subsequently transferred to a different hospital for rehabilitation, where he had an uneventful course with no recurrence of acidosis or infectious complications. He was subsequently discharged with home support services and instructions for outpatient follow up.

## 5 DISCUSSION

**FIGURE 3:**
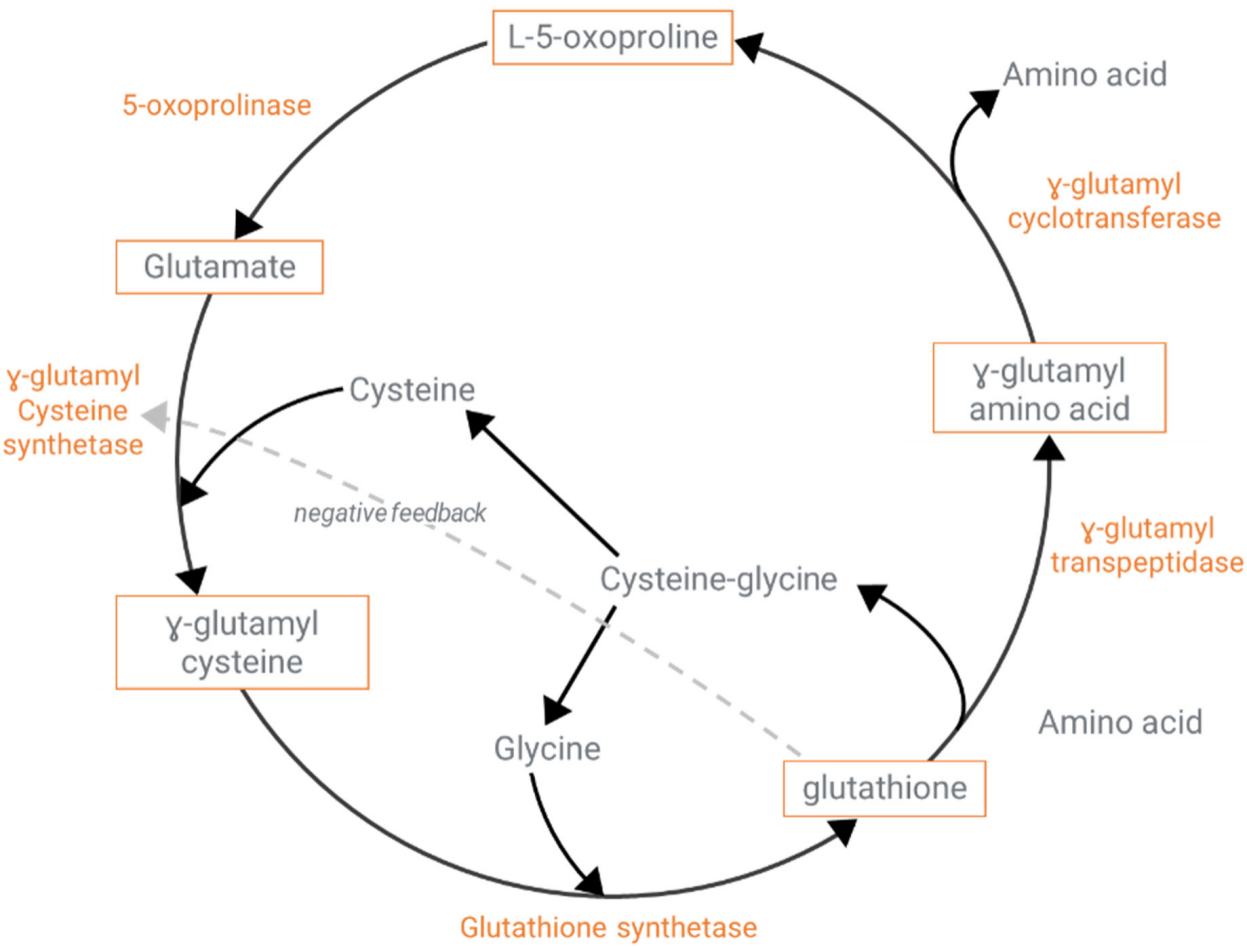
The ɣ‐glutamyl cycle.

Pyroglutamic acid (5‐Oxoproline) is an organic acid metabolite of the glutathione cycle, where it is involved in the synthesis of glutathione (Figure 3). Pyroglutamic acid is synthesized from γ‐glutamic amino acids by the enzyme γ‐glutamic cyclotransferase. High levels of glutathione provide negative feedback into the synthesis of pyroglutamic acid.^3^ Consequently, glutathione‐depleted states can result in the overproduction and accumulation of pyroglutamic acid. The gradual accumulation of pyroglutamic acid in turn causes PGA.^4^ Conditions associated with glutathione depletion and decreased pyroglutamic acid clearance confer a risk of developing PGA. Such conditions include sepsis, certain medications (e.g., paracetamol and flucloxacillin), liver disease, renal impairment, and malnutrition.^4^

PGA is a rare cause of anion gap metabolic acidosis. The diagnosis of PGA is one of exclusion and is an important differential in the investigation of HAGMA. This case highlights the importance of having a structured approach and workup of HAGMA. When calculating the anion gap, correcting for low serum albumin is essential, especially when a previous review of cases demonstrated that hypoalbuminemia can be in seen up to a third of cases.^5^ Failure to do so might result in misinterpretation of the anion gap. In terms of clinical presentation, nonspecific symptoms of nausea, vomiting, and altered mental status may be present.^6^ The clinical history might also provide invaluable clues. In a review of 100 cases of pyroglutamic acidosis (mean reported age of 55), the most commonly identified factors included paracetamol use (93%), female gender (73%), raised creatinine (34%), and flucloxacillin use (32%). Interestingly, vigabatrin use was only seen in 3% of the cases, despite being an often‐cited contributor to PGA.^5^ In this case, while the patient did have significant risk factors such as recent paracetamol and flucloxacillin use, other common risk factors such as female gender and impaired renal function were absent. This stresses the importance of maintaining awareness of the condition even in the absence of classic risk factors. Clinical suspicion for PGA should also be raised in worsening HAGMA despite clinical improvement in conditions such as sepsis, especially in the setting of down trending or normal lactate levels. Markers such as osmolar gap, lactate, and ketones are usually normal or only mildly elevated in cases of PGA.^7^ Besides blood gases and other routine biochemical workup, serum or urine pyroglutamic acid levels can be directly measured if clinical suspicion is high.^4^ However, treatment should not be delayed in the absence of confirmatory results, as biochemical analyses of pyroglutamic acid levels can take time.

The main management of PGA is discontinuation of contributing medications.^8^ Notably in this case, it was decided with specialist input that flucloxacillin was to be trialed at a reduced dose frequency despite the diagnosis of PGA. This differs from other cases where flucloxacillin was immediately ceased upon diagnosis.^9^, ^10^ Worsening of HAGMA despite dose reduction suggests that cessation and swapping of antibiotics is the recommended course to treat PGA. Besides discontinuation, administration of N‐acetylcysteine (NAC) may be considered in severe cases of acidosis—this is based on the notion that replenishing glutathione should re‐establish the negative feedback loop governing polyglutamic acid production.^3^ Individual reports have reported good response to NAC.^8^, ^11^ Notably, one case highlighted the persistence of anion gap acidosis despite paracetamol discontinuation, with immediate resolution seen only after the administration of NAC.^11^ However, no trials or large cohort studies have been conducted to determine the efficacy of NAC. Bicarbonate is also another commonly used treatment option. While clinical guidelines do not recommend the usage of bicarbonate in other causes of HAGMA such as diabetic ketoacidosis and lactic acidosis, no trials have been conducted on the efficacy of bicarbonate in PGA.^12^ In addition, previous reports have almost always used bicarbonate in conjunction with medication cessation and/or NAC, making it difficult to assess its efficacy. However, a report on two pediatric patients with inborn error in glutathione metabolism (resulting in pyroglutamic acid overproduction) demonstrated stabilization of acidosis with daily bicarbonate in the absence of other interventions.^13^ Another factor to consider is the presence of other causes of acidosis that have a stronger indication for bicarbonate, such as NAGMA secondary to gastrointestinal losses.^12^ In cases refractory to treatment, hemodialysis has been reported to accelerate the clearance of pyroglutamic acid followed by sustained clinical improvement.^4^, ^10^

Further studies are needed to clarify the extent to which medication discontinuation, NAC, and bicarbonate help treat pyroglutamic acidosis. Larger studies may also assist in better characterizing risk factors for pyroglutamic acidosis. For example, based on anecdotal evidence and observations of other case reports, patients who developed pyroglutamic acidosis are often on non‐vigabatrin anticonvulsant drugs such as levetiracetam, topiramate, and sodium valproate.^8^, ^9^, ^10^ Better characterization of etiology and risk factors could lead to the development of risk scores and clinical alerts, which could assist clinicians in screening and prognosticating this condition. This is supported by a retrospective study based on a 2‐year study period of approximately 53,000 admissions, in which a simulated clinical screening of acidosis (pH of ≤7.35) and simultaneous use of paracetamol and flucloxacillin was able to accurately detect all cases of PGA with only 25 alarm notifications.^14^

## 6 CONCLUSION

This report highlights PGA as an important differential for HAGMA, particularly when the presence of factors that disrupt glutathione metabolism are present. In addition, the incidental and asymptomatic nature of the presentation emphasizes the importance of having a systematic approach to HAGMA. This includes conducting a thorough clinical evaluation and laboratory analyses to ensure timely and accurate diagnosis. Additionally, this case also highlights the evidence behind management options for PGA, including medication discontinuation, NAC, bicarbonate, and dialysis.

## AUTHOR CONTRIBUTIONS

**Aaron Yee Shuen See:** Conceptualization; data curation; formal analysis; investigation; writing – original draft; writing – review and editing. **Ashton Zheng‐Boon Lau:** Conceptualization; data curation; formal analysis; investigation; writing – original draft; writing – review and editing. **Srivathsan Thiruvengadam:** Conceptualization; data curation; formal analysis; investigation; supervision; writing – original draft; writing – review and editing.

## FUNDING INFORMATION

No funding was received for this study.

## CONFLICT OF INTEREST STATEMENT

Aaron Yee Shuen See, Asthon Zheng‐Boon Lau, Srivathsan Thiruvengadam have none to declare.

## CONSENT

A written informed consent was obtained by the patient's public guardian and is available for review by the Editor‐in‐Chief of this journal. In addition, diligent care has been taken to ensure that the International Committee of Medical Journal Editors (ICMJE) recommendations have been adhered to with respect to preserving the patient's privacy and identity.

## Data Availability

All accessed data and literature relevant to this study are included in this article.

## References

[1] Mehta AN , Emmett JB , Emmett M . GOLD MARK: an anion gap mnemonic for the 21st century. Lancet. 2008;372(9642):892. 18790311 10.1016/S0140-6736(08)61398-7

[2] Berend K , de Vries AP , Gans RO . Physiological approach to assessment of acid‐base disturbances. N Engl J Med. 2015;372(2):195. doi:10.1056/NEJMc1413880 25564913

[3] Emmett M . Acetaminophen toxicity and 5‐oxoproline (pyroglutamic acid): a tale of two cycles, one an ATP‐depleting futile cycle and the other a useful cycle. Clin J Am Soc Nephrol. 2014;9(1):191‐200. 24235282 10.2215/CJN.07730713 PMC3878708

[4] Alhourani HM , Kumar A , George LK , Sarwar T , Wall BM . Recurrent pyroglutamic acidosis related to therapeutic acetaminophen. Am J Med Sci. 2018;355(4):387‐389. 29661353 10.1016/j.amjms.2017.08.001

[5] Stewart GW . Pyroglutamate acidosis 2023. A review of 100 cases. Clin Med (Lond). 2024;24(2):100030. doi:10.1016/j.clinme.2024.100030 38431210 PMC11091441

[6] Venkataraman SS , Regone R , Ammar HM , Govindu RR . Pyroglutamic acidemia: an underrecognized and underdiagnosed cause of high anion gap metabolic acidosis—a case report and review of literature. Cureus. 2019;11(7):e5229. 31565630 10.7759/cureus.5229 PMC6758980

[7] Raibman Spector S , Mayan H , Loebstein R , et al. Pyroglutamic acidosis as a cause for high anion gap metabolic acidosis: a prospective study. Sci Rep. 2019;9(1):3554. 30837497 10.1038/s41598-019-39257-4 PMC6400893

[8] Serpa MJ , Falcão L , Franco S , Repolho D , Ferreira NR . Metabolic acidosis due to pyroglutamic acid. Eur J Case Rep Intern Med. 2018;5(10):000949. 30755980 10.12890/2018_000949 PMC6346886

[9] Zand Irani A , Borchert G , Craven B , Gibbons H . Flucloxacillin and paracetamol induced pyroglutamic acidosis. BMJ Case Rep. 2021;14(1):e237536. 10.1136/bcr-2020-237536 PMC7798777 33419747

[10] Luyasu S , Wamelink MM , Galanti L , Dive A . Pyroglutamic acid‐induced metabolic acidosis: a case report. Acta Clin Belg. 2014;69(3):221‐223. 24694265 10.1179/2295333714Y.0000000022

[11] Hundemer GL , Fenves AZ . Acquired 5‐oxoproline acidemia successfully treated with N‐acetylcysteine. Proc (Bayl Univ Med Cent). 2017;30(2):169‐170. doi:10.1080/08998280.2017.11929570 28405069 PMC5349815

[12] Wardi G , Holgren S , Gupta A , et al. A review of bicarbonate use in common clinical scenarios. J Emerg Med. 2023;65(2):e71‐e80. doi:10.1016/j.jemermed.2023.04.012 37442665 PMC10530341

[13] Larsson A , Zetterström R , Hagenfeldt L , Andersson R , Dreborg S , Hörnell H . Pyroglutamic aciduria (5‐oxoprolinuria), an inborn error in glutathione metabolism. Pediatr Res. 1974;8(10):852‐856. doi:10.1203/00006450-197410000-00007 4415411

[14] Berbee JK , Lammers LA , Krediet CTP , Fischer JC , Kemper EM . Metabolic acidosis caused by concomitant use of paracetamol (acetaminophen) and flucloxacillin? A case report and a retrospective study. Eur J Clin Pharmacol. 2017;73(11):1459‐1465. doi:10.1007/s00228-017-2311-6 28782093 PMC5662679

